# Elevated hsa‐miR‐590‐3p expression down‐regulates HMGB2 expression and contributes to the severity of IgA nephropathy

**DOI:** 10.1111/jcmm.14582

**Published:** 2019-09-26

**Authors:** Yaling Zhai, Yuanyuan Qi, Xiaoqing Long, Yanna Dou, Dong Liu, Genyang Cheng, Jing Xiao, Zhangsuo Liu, Zhanzheng Zhao

**Affiliations:** ^1^ Department of Nephrology, The First Affiliated Hospital of Zhengzhou University The Renal Research Institution of Zhengzhou University Zhengzhou China

**Keywords:** hsa‐miR‐590‐3p, HMGB2, IgA nephropathy

## Abstract

Peripheral blood mononuclear cells (PBMCs) play important roles in the pathogenesis of IgA nephropathy (IgAN). Our study aimed to provide a deep understanding of IgAN and focused on the dysregulation of hsa‐miR‐590‐3p and its target gene *HMGB2* in PBMCs. Three gene expression profile datasets (GSE14795, GSE73953 and GSE25590) were downloaded from the GEO database. The DEGs (differentially expressed genes)‐miRNA network that was associated with IgAN was constructed by Cytoscape, and HMGB2 and hsa‐miR‐590‐3p were selected for further exploration. The dual‐luciferase reporter system was utilized to verify their interaction. Then, the expression levels of HMGB2 and hsa‐miR‐590‐3p in PBMCs were detected by qPCR in another cohort, and the correlation of their expression levels with the clinical pathological manifestations and serum Gd‐IgA1(galactose‐deficient IgA1) levels was also investigated. *HMGB2* was identified as the target gene of hsa‐miR‐590‐3p. Furtherly, the elderly patients had higher HMGB2 expression levels than the expression levels of the younger patients. As the serum creatinine, serum BUN levels increased, the expression of HMGB2 decreased; Besides, the HMGB2 expression was positively correlated with serum complement 3(C3) levels, and it also had a negative correlation with the diastolic blood pressure, but not reach statistical significance. What is more, both hsa‐miR‐590‐3p and HMGB2 expression had a slight correlation tendency with serum Gd‐IgA1 levels in the whole population. In conclusion, HMGB2, the target gene of hsa‐miR‐590‐3p, was identified to correlate with the severity of IgAN, and this provides more clues for the pathogenesis of IgAN.

## 1 INTRODUCTION

Immunoglobulin A nephropathy (IgAN) is the most common form of primary glomerulonephritis worldwide and is characterized by predominant IgA deposition in the mesangial area. Approximately, one‐third of patients with IgAN progress to the end stage of renal disease (ESRD) within two decades and need renal replacement therapy (RRT), which brings heavy health and eco‐social burdens.^1^, ^2^ However, the exact pathogenesis of IgAN has remained obscure until now. IgAN is a multifactorial disease, and both environmental and genetic effects contribute to it. Recently, the multihit pathogenesis model of IgAN has been widely accepted,^3^ which indicates that circulating galactose‐deficient IgA1 is the cause. The contribution of galactose‐deficient IgA1 to IgAN pathogenesis has been validated in many studies.^4^, ^5^, ^6^ Moreover, the presence of galactose‐deficient IgA1(Gd‐IgA1) in the glomerular deposits of patients with IgAN has been proven by immunohistochemical staining using the galactose‐deficient IgA1‐specific monoclonal antibody KM55,^7^, ^8^ which reinforces the important role that galactose‐deficient IgA1 plays in IgAN.

Previous studies have shown that the aberrant deposition of glycosylated IgA1 in the renal mesangial area was from circulation.^9^, ^10^ The production of IgA1, including the O‐glycosylation status of IgA1, was regulated not only by B cells but also by some cytokines secreted by T cells, dendritic cells, and monocytes,^11^, ^12^ all of which make up the majority of peripheral blood mononuclear cells (PBMCs). Moreover, previous studies have identified many key proteins that are involved in the O‐glycosylation of IgA1, including APRIL and BAFF, and all of these proteins were expressed in PBMCs,^13^, ^14^, ^15^ indicating that PBMCs are a whole cell population and that further investigation is needed.

MicroRNAs (miRNAs) are a class of single‐stranded, short RNA molecules that down‐regulate gene expression by binding to specific sites within the 3′ untranslated regions (UTRs) of mRNAs to promote mRNA degradation or to interrupt translation processes.^16^ MiRNAs can exist in the cell or can be secreted selectively out of the cell, with the regulatory function of gene expression and cell‐to‐cell communication. In recent years, great progress regarding miRNAs has been achieved in the field of nephrology. Many differentially expressed miRNAs in several kinds of human samples were identified as biomarkers or participants in IgAN pathogenesis,^17^, ^18^, ^19^, ^20^, ^21^, ^22^ indicating the important pathophysiological role of miRNAs in IgAN.

In the present study, we summarized and reanalysed the previously reported microarray data of PBMCs in IgAN to explore the miRNAs and target genes that are associated with IgAN.

## 2 MATERIALS AND METHODS

### 2.1 Microarray data preprocessing

**Figure 1:**
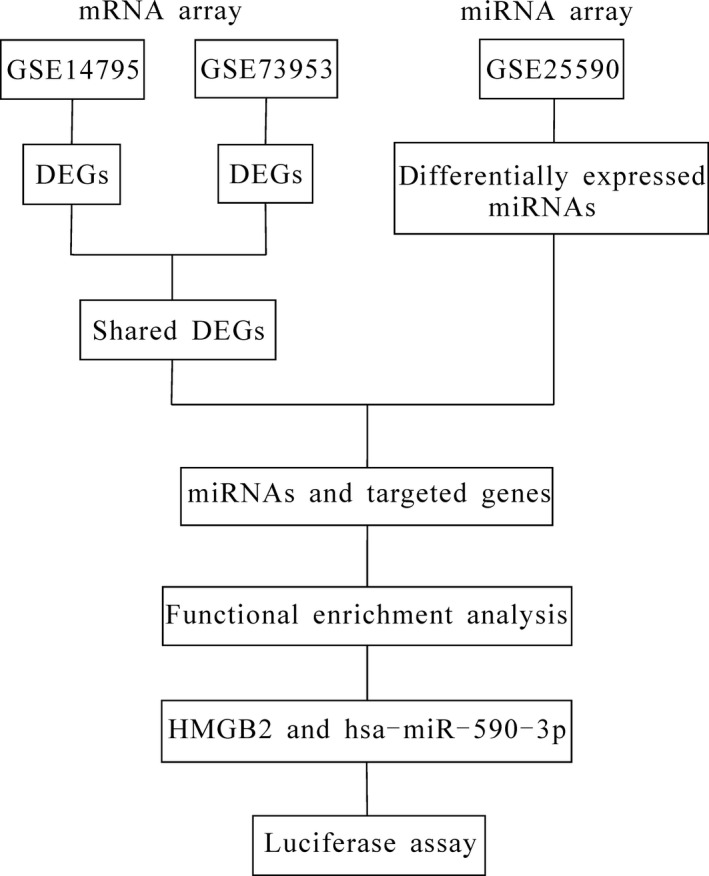
Flowchart of the analysis process

All microarray data derived from PBMCs in IgAN were searched in the Gene Expression Omnibus database (http://www.ncbi.nlm.nih.gov/geo/). We obtained three datasets, including two mRNA arrays, GSE14795^23^ and GSE73953,^24^ and one miRNA array, GSE25590.^20^ The data of patients with IgAN and controls were extracted in GSE14795 and GSE73953 for subsequent analysis (the data analysis pipeline is shown in Figure 1). The samples included in each dataset and the corresponding annotations for the array platform are listed in Table 1. All data were Log_2_ transformed to achieve normality. In addition, data normalization was performed with the linear models for the microarray data (limma, http://www.R-project.org) package in R. Principal component analysis (PCA) and clustering were also performed for the data quality control. The samples that did not reach the quality control standards were excluded, as shown in Table 1.

**Table 1:** Information on the three gene expresssion profile dataset

	Before nomalization		After normalization		Platforms
	IgAN sample count	Control sample count	IgAN sample count	Control sample count	
GSE14795	12	8	8	6	Affymetrix Human Genome U133A Array
GSE73953	15	16 (pooled)	15	16 (poold)	Agilent‐014850 Whole Human Genome Microarray 4x44K G4112F
GSE25590	7	7	4	3	GPL7731 Agilent‐019118 Human miRNA Microarray 2.0

### 2.2 DEG analysis and functional enrichment analysis

First, the samples in GSE14795 were used as the discovery cohort, and GSE73953 was used as the validation cohort to obtain the shared differentially expressed genes (DEGs). Second, the target genes of the differentially expressed miRNAs of GSE25590 were predicted using 4 miRNA databases:MiRDB,^25^ Tarbase,^26^ miRTarBase,^27^ and TargetScan.^28^ Finally, we merged the above results together and obtained the gene‐miRNA network. QuickGO,^29^ a web‐based tool for gene ontology searching, was utilized for identifying the enriched functions in ‘shared DEGs’, and the Kyoto Encyclopedia of Genes and Genomes (KEGG),^30^ a reference resource for gene and protein annotation, was used to assign ‘shared DEGs’ to specific pathways. *P* < .05 was used as the threshold value.

### 2.3 IgAN‐associated gene‐miRNA network analysis

The Search Tool for the Retrieval of Interacting Genes (STRING) database^31^ (http://string-db.org) was used to provide information on the different proteins, including the predicted and experimental interactions and the direct (physical) and indirect (functional) interactions of the proteins. The ‘IgAN‐associated genes and relative miRNAs’ were mapped into the network. Cytoscape software,^32^ a software for the integrated models of biomolecular interaction networks, was used to construct the IgAN‐associated gene‐miRNA network.

### 2.4 Luciferase reporter assays

HMGB2 and hsa‐miR‐590‐3p were chosen for further validation of the IgAN‐associated gene‐miRNA network. The dual‐luciferase reporter system was applied to verify the interaction between hsa‐miR‐590‐3p and its target gene *HMGB2*. Briefly, the 3′UTR sequences of the *HMGB2* gene were amplified from genomic DNA and were subcloned directly downstream of the Renilla luciferase gene in the pmiR‐GLO vector. A mutant version of the 3′UTR sequences of *HMGB2* in the ‘seed region’ was also generated as the mutant control. Both constructs were verified by DNA sequencing. To determine whether hsa‐miR‐590‐3p could bind to the 3′UTR sequences of the *HMGB2* gene, HEK293T cells were seeded into 24‐well plates. After 24 hours, the cells were cotransfected with 25 nmol of hsa‐miR‐590‐3p mimics or the negative control (NC) mimics, along with 800 ng per well of pmiR‐GLO‐3’UTR‐HMGB2 wild‐type construct (pmiR‐GLO‐3’UTR‐HMGB2^wt^) or the mutant version (pmiR‐GLO‐3’UTR‐HMGB2^mu^) performed with Lipofectamine 2000 (Invitrogen) according to the manufacturer's protocol. After 48 hours of culture, the cells were lysed, and the firefly and Renilla luciferase activities were quantified using the Dual‐Luciferase Reporter Assay System (Promega). The reporter activity of each well was expressed as the relative luciferase expression normalized to the Renilla activity.

### 2.5 Study population

Thirty‐seven primary IgAN patients diagnosed at The First Affiliated Hospital of Zhengzhou University were recruited between February 2018 and June 2018. The granular deposition of IgA in the glomerular mesangium by immunofluorescence detection and the deposition of electron‐dense material in the mesangium by ultra‐structural examination confirmed the diagnosis of IgAN. Patients were excluded if the following items are met: (a) patients with Henoch‐Schonlein Purpura, systemic lupus erythematosus or chronic hepatic diseases; (b) patients had used corticosteroids or immunosuppressors; (c) IgAN was suspected secondary to other diseases. At the same time, nine age‐ and gender‐matched healthy controls in the physical examination center of this hospital were recruited, after carefully checking the examination results. For each recruited individual, 10‐ml peripheral blood was obtained, for patients on the morning of renal biopsy and for controls on the day of recruitment. RNA extraction was performed to determine the expression of *HMGB2* and hsa‐miR‐590‐3p, the plasma samples were divided into aliquots and stored in −80°C for subsequent detection of Gd‐IgA1 protein levels. The clinical and pathological data at the time of renal biopsy were collected from medical records for further analysis. Notably, all the patients did not receive any special treatment before, such as immunosuppressants, ACEI/ARB.

The Medical Ethics Committee of The First Affiliated Hospital of Zhengzhou University approved the study protocol, and informed written consent was obtained from all participants.

### 2.6 Detection of the expression of HMGB2 and hsa‐miR‐590‐3p

Peripheral blood mononuclear cells (PBMCs) were prepared by density gradient centrifugation performed with Ficoll‐Paque Plus (GE). After cell isolation, the total RNA was extracted using the commercial TRIzol Reagent (Invitrogen). The cDNA for miRNA and mRNA detection was synthesized from 1 μg total RNA using the Reverse Transcription System (for miRNA: TIANGEN, Beijing, China; for mRNA: Promega, Wisconsin, USA) and was stored at −20°C for the following amplification. The expression levels of HMGB2 and hsa‐miR‐590‐3p were measured by semiquantitative reverse transcription‐PCR performed with AceQ^®^ qPCR SYBR^®^ Green Master Mix (Takara), and this experiment was performed on an Applied Biosystem 7500 Real‐Time PCR System (the primers are shown in Table 2). U6 snRNA and GAPDH were used for normalization. The expression fold change between the patients and the controls was expressed by the 2^−ΔΔCT^ method.

**Table 2:** The primer pairs for HMGB2, GAPDH and has‐mir‐590‐3p

	Sequence	
HMGB2	F	5′‐GTGGCCTAGCTCGTCAAGTT‐3′
	R	5′‐GCGTACGAGGACATTTTGCC‐3′
GAPDH	F	5′‐GGGAAACTGTGGCGTGAT‐3′
	R	5′‐GAGTGGGTGTCGCTGTTGA‐3′
hsa‐miR‐590‐3p	F	5′‐TAATTTTATGTATAAGCTAGT‐3′
	R	5′‐GTGCAGGGTCCGAGGT‐3′

### 2.7 Detection of plasma Gd‐IgA1 levels

Plasma Gd‐IgA1 levels were detected performed with a commercial enzyme‐linked immunosorbent assay (ELISA) kit according to the manufacturer's specifications (IBL).

### 2.8 Statistical analysis

Statistical analyses were performed with SPSS software (version 16.0; SPSS). For continuous variables, data with normal distribution were expressed as the mean ± SD and were compared by an independent‐sample *t* test, and Pearson Correlation was used if need; the other data were expressed as the median (first quartile and third quartile) and were analysed by the Mann‐Whitney *U* test, and the Spearman Correlation was utilized for the correlation analysis. A two‐tailed *P*‐value <.05 was considered statistically significant. The graphs were plotted with GraphPad Prism.

## 3 RESULTS

### 3.1 Screened miRNAs and target genes associated with IgAN

First, 894 DEGs (168 up‐regulated and 726 down‐regulated genes) and 2668 DEGs (355 up‐regulated and 2313 down‐regulated genes) were obtained from GSE14795 and GSE73953, respectively. Then, 129 ‘shared DEGs’ were obtained; finally, 38 differentially expressed miRNAs in the GSE25590 dataset were merged with 129 ‘shared DEGs’ from GSE14795 and GSE73953, and 7 miRNAs and 19 target genes were identified (as shown in Table 3). Then, the genes were constructed with functional and pathway enrichment analysis by QuickGO and KEGG (shown in Table 4). We set the gene number above 6 for each GO term and a p‐value less than 0.01 as the cutoff values, and we obtained 25 GO terms. Regarding the KEGG pathway, 2 pathways were identified with *P* < .05, but each included only 1 gene.

**Table 3:** 7miRNAs and 19 targeted genes associated with IgAN

Gene symbol	Fold change (Log2 transformed)	microRNA
*ARID4A*	−1.65	hsa‐miR‐519c‐3p
*CETN3*	−1.75	hsa‐miR‐520b
*CNOT3*	−1.21	hsa‐miR‐920
*GCC2*	−1.29	hsa‐miR‐590‐3p
*HABP4*	−1.76	hsa‐miR‐519c‐3p
*HMGB2*	−1.22	hsa‐miR‐590‐3p
*IFIT5*	−1.13	hsa‐miR‐384
*JMJD1C*	−1.85	hsa‐miR‐648
*MAN1A1*	−1.29	hsa‐miR‐590‐3p
*MAPK6*	−1.37	hsa‐miR‐384
*NAMPT*	−1.12	hsa‐miR‐590‐3p
*NPTN*	−1.29	hsa‐miR‐873
*RAB1A*	−1.57	hsa‐miR‐648
*RASA1*	−1.17	hsa‐miR‐648
*RBM26*	−1.33	hsa‐miR‐384
*TGFA*	−1.65	hsa‐miR‐384
*TMEM57*	−1.29	hsa‐miR‐384
*TUBGCP3*	−1.42	hsa‐miR‐873
*ZFR*	−1.09	hsa‐miR‐384

**Table 4:** GO and KEGG enrichment analysis results for DEGs identified in the three microarray

Description	gene count	Pvalue	Genes
GO terms			
GO:0044699‐single‐organism process	18	1.00 × 10^‐3^	*ZFR;TMEM57;MAN1A1;HABP4;IFIT5;TGFA;CETN3;GCC2;JMJD1C;NAMPT;RASA1;MAPK6;RAB1A;ARID4A;TUBGCP3;HMGB2;NPTN;CNOT3*
GO:0044707‐single‐multicellular organism process	13	4.07 × 10^‐6^	*ZFR;TMEM5;HABP4;TGFA;JMJD1C;NAMPT;RASA1;MAPK6;RAB1A;ARID4A;HMGB2;NPTN;CNOT3*
GO:0032501‐multicellular organismal process	13	7.82 × 10^‐6^	*ZFR;TMEM57;HABP4;TGFA;JMJD1C;NAMPT;RASA1;MAPK6* *;RAB1A;ARID4A;HMGB2;NPTN;CNOT3*
GO:0048856‐anatomical structure development	11	5.60 × 10^‐5^	*ZFR;TMEM57;;NAMPT;RASA1;MAPK6* *;RAB1A;ARID4A;HMGB2;NPTN;CNOT3*
GO:0044767‐single‐organism developmental process	11	1.13 × 10^‐4^	*ZFR;TMEM57;TGFA;NAMPT;RASA1;MAPK6* *;RAB1A;ARID4A;HMGB2;NPTN;CNOT3*
GO:0032502‐developmental process	11	1.32 × 10^‐4^	*ZFR;TMEM57;TGFA;NAMPT;RASA1;MAPK6* *;RAB1A;ARID4A;HMGB2;NPTN;CNOT3*
GO:0060255‐regulation of macromolecule metabolic process	11	1.57 × 10^‐3^	*HABP4;TGFA;JMJD1C;NAMPT;RASA1;MAPK6* *;RAB1A;ARID4A;HMGB2;NPTN;CNOT3*
GO:0019222‐regulation of metabolic process	11	6.12 × 10^‐3^	*HABP4;TGFA;JMJD1C;NAMPT;RASA1;MAPK6* *;RAB1A;ARID4A;HMGB2;NPTN;CNOT3*
GO:0007275‐multicellular organism development	10	1.02 × 10^‐4^	*ZFR;TMEM57;TGFA;NAMPT;RASA1;MAPK6* *;ARID4A;HMGB2;NPTN;CNOT3*
GO:0048518‐positive regulation of biological process	10	5.60 × 10^‐4^	*IFIT5;TGFA;NAMPT;RASA1;MAPK6* *;RAB1A;ARID4A;HMGB2;NPTN;CNOT3*
GO:0016043‐cellular component organization	10	5.45 × 10^‐3^	*CETN3;GCC2;JMJD1C;RASA1;MAPK6* *;RAB1A;ARID4A;TUBGCP3;HMGB2;NPTN*
GO:0080090‐regulation of primary metabolic process	10	6.26 × 10^‐3^	*HABP4;TGFA;JMJD1C;NAMPT;RASA1;RAB1A;ARID4A;HMGB2;NPTN;CNOT3*
GO:0071840‐cellular component organization or biogenesis	10	6.85 × 10^‐3^	*CETN3;GCC2;JMJD1C;RASA1;MAPK6* *;RAB1A;ARID4A;TUBGCP3;HMGB2;NPTN*
GO:0031323‐regulation of cellular metabolic process	10	6.93 × 10^‐3^	*HABP4;TGFA;JMJD1C;NAMPT;RASA1;RAB1A;ARID4A;HMGB2;NPTN;CNOT3*
GO:0048522‐positive regulation of cellular process	9	5.74 × 10^‐4^	*IFIT5;TGFA;NAMPT;MAPK6* *;RAB1A;ARID4A;HMGB2;NPTN;CNOT3*
GO:0048731‐system development	8	1.59 × 10^‐3^	*TMEM57;TGFA;NAMPT;RASA1;MAPK6* *;ARID4A;HMGB2;NPTN*
GO:0006996‐organelle organization	8	4.66 × 10^‐3^	*CETN3;GCC2;JMJD1C;RASA1;RAB1A;ARID4A;TUBGCP3;HMGB2*
GO:0006464‐cellular protein modification process	8	9.25 × 10^‐3^	*MAN1A1;TGFA;JMJD1C;RASA1;MAPK6* *;RAB1A;ARID4A;NPTN*
GO:0036211‐protein modification process	8	9.25 × 10^‐3^	*MAN1A1;TGFA;JMJD1C;RASA1;MAPK6* *;RAB1A;ARID4A;NPTN*
GO:0048468‐cell development	7	9.85 × 10^‐5^	*RASA1;MAPK6* *;RAB1A;ARID4A;HMGB2;NPTN;CNOT3*
GO:1902589‐single‐organism organelle organization	7	2.16 × 10^‐3^	*CETN3;GCC2;RASA1;RAB1A;ARID4A;TUBGCP3;HMGB2*
GO:0009893‐positive regulation of metabolic process	7	3.75 × 10^‐3^	*TGFA;NAMPT;RASA1;RAB1A;ARID4A;HMGB2;NPTN*
GO:0030154‐cell differentiation	7	3.83 × 10^‐3^	*RASA1;MAPK6* *;RAB1A;ARID4A;HMGB2;NPTN;CNOT3*
GO:0065008‐regulation of biological quality	7	4.32 × 10^‐3^	*HABP4;JMJD1C;RASA1;RAB1A;ARID4A;HMGB2;NPTN*
GO:0048869‐cellular developmental process	7	7.37 × 10^‐3^	*RASA1;MAPK6* *;RAB1A;ARID4A;HMGB2;NPTN;CNOT3*
**KEGG pathways**			
hsa00760‐Nicotinate and nicotinamide metabolism	1	2.94 × 10^‐2^	*NAMPT*
hsa00510‐N‐Glycan biosynthesis	1	4.76 × 10^‐2^	*MAN1A1*

**Figure 2:**
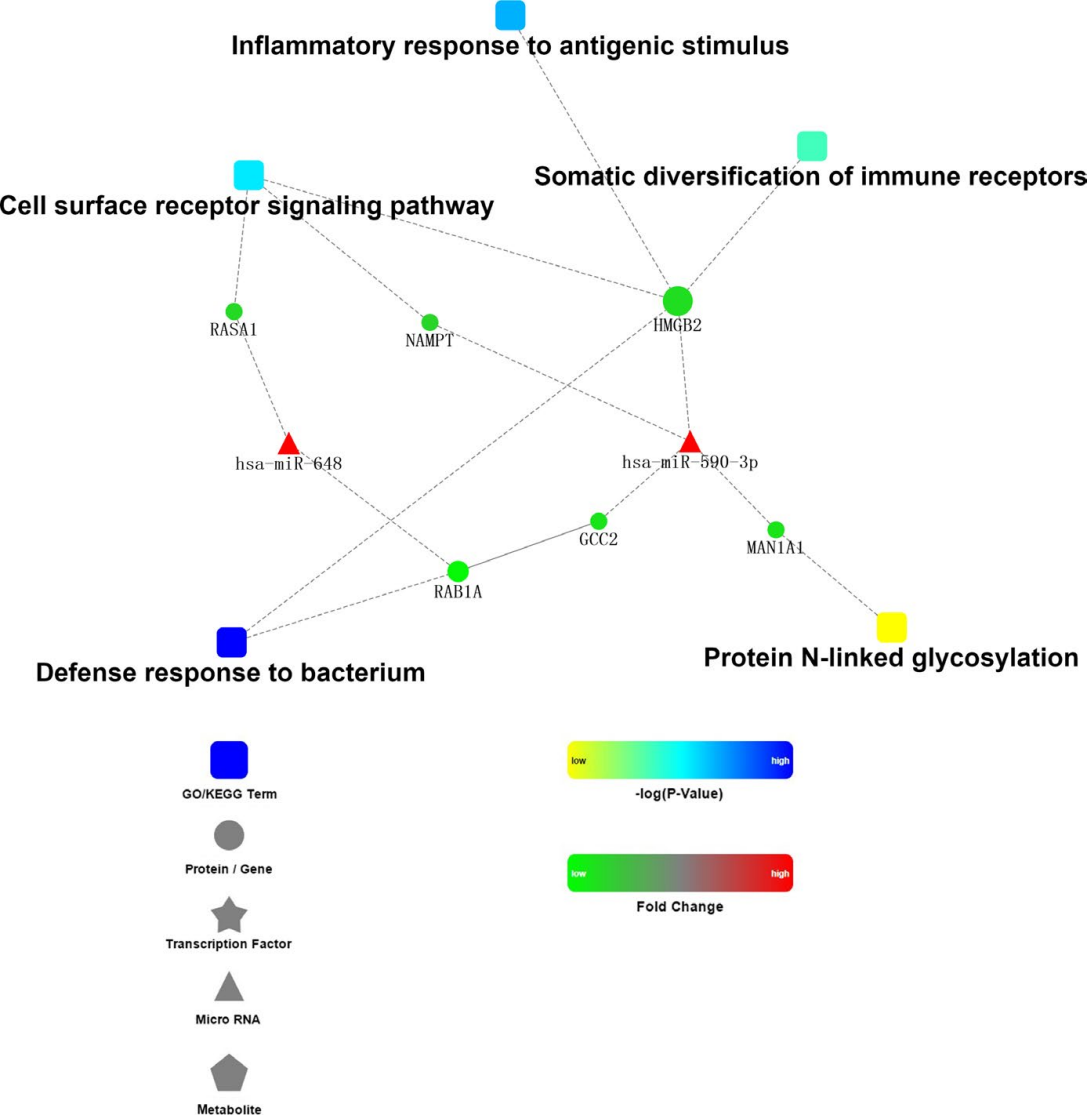
IgAN‐associated gene‐miRNA network

IgAN is a multifactorial disease with unclear pathogenesis. Previous studies have demonstrated that immune system disorders, susceptible genes and inflammation are the contributing factors. The IgAN‐associated gene‐miRNA network was constructed using Cytoscape (shown in Figure 2). In total, five enriched pathways containing six genes*, HMGB2, MAN1A1, NAMPT, NPTN, RASA1,* and *GCC2* and 2 miRNAs, hsa‐miR‐590‐3p and hsa‐miR‐648, were identified to be potentially involved in the pathogenesis of IgAN. From the IgAN‐associated gene‐miRNA network, *HMGB2* was the core and most weighted gene among the 6 genes because of its significantly different expression in the two mRNA datasets (GSE14795 and GSE73953) and because of its involvement in four IgAN‐associated pathways (inflammatory response to antigenic stimulus, defense response to bacteria, somatic diversification of immune receptors and cell surface receptor signalling pathway).^3^, ^33^, ^34^ Therefore, *HMGB2* was chosen for further validation.

### 3.2 *HMGB2* is a direct target of hsa‐miR‐590‐3p

**Figure 3:**
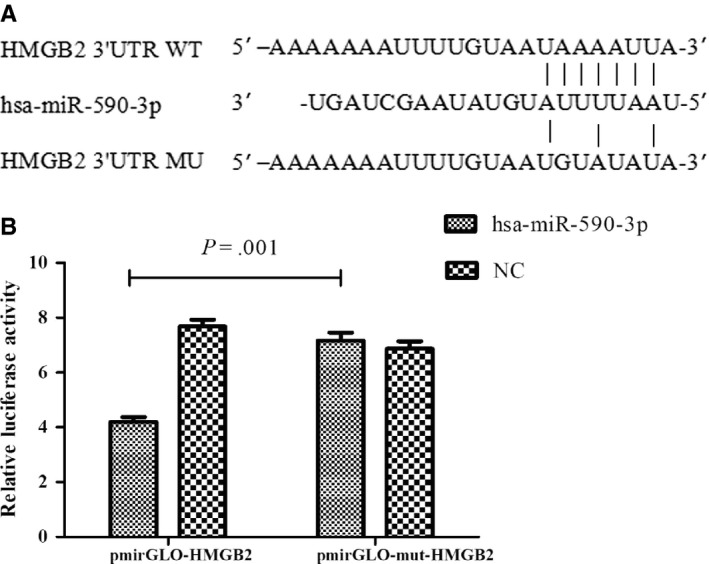
HMGB2 is the target gene of hsa‐miR‐590‐3p (a) Binding site of hsa‐miR‐590‐3p in the HMGB2 wild‐type (WT) or mutation (MU) 3′‐UTR. (b) Luciferase reporter assays were performed to verify the binding of hsa‐miR‐590‐3p in 3′‐UTR of HMGB2. HEK293T cells cotransfected with hsa‐mir‐590‐3p mimics and pmiR‐GLO‐3’UTR‐HMGB2^wt^ displayed significantly reduced luciferase activity compared with those cotransfected with NC and pmiR‐GLO‐3’UTR‐HMGB2^mu^ (4.20 ± 0.30 vs 7.16 ± 0.51, *P* = .001). While the luciferase activity of cells cotransfected with hsa‐mir‐590‐3p mimics and pmiR‐GLO‐3’UTR‐HMGB2^mu^ displayed no significant changes compared with cells cotransfected with NC and pmiR‐GLO‐3’UTR‐HMGB2^mu^

The 3’UTR of *HMGB2* was predicted to have a conserved binding site for hsa‐miR‐590‐3p (363th‐370th bp), as shown in Figure 3A. To further confirm *HMGB2* as the putative target of hsa‐miR‐590‐3p, an hsa‐miR‐590‐3p mimic or a negative control (NC) sequence was cotransfected with constructs containing the wild‐type or mutant *HMGB2* 3’UTR into HEK293T cells. As shown in Figure 3B, the HEK293T cells that were cotransfected with hsa‐miR‐590‐3p mimics and pmiR‐GLO‐3’UTR‐*HMGB2* ^wt^ displayed significantly reduced luciferase activity levels compared with those cotransfected with NC and pmiR‐GLO‐3’UTR‐*HMGB2* ^mu^ (4.20 ± 0.30 vs 7.16 ± 0.51, *P* = .001). The luciferase activity of cells cotransfected with hsa‐miR‐590‐3p mimics and pmiR‐GLO‐3’UTR‐*HMGB2* ^mu^ displayed no significant changes compared with the luciferase activity of cells cotransfected with NC and pmiR‐GLO‐3’UTR‐*HMGB2* ^mu^, indicating that hsa‐miR‐590‐3p binds to the 3’UTR of *HMGB2* and down‐regulates its expression.

### 3.3 Increased hsa‐miR‐590‐3p expression and decreased HMGB2 expression in IgAN

**Figure 4:**
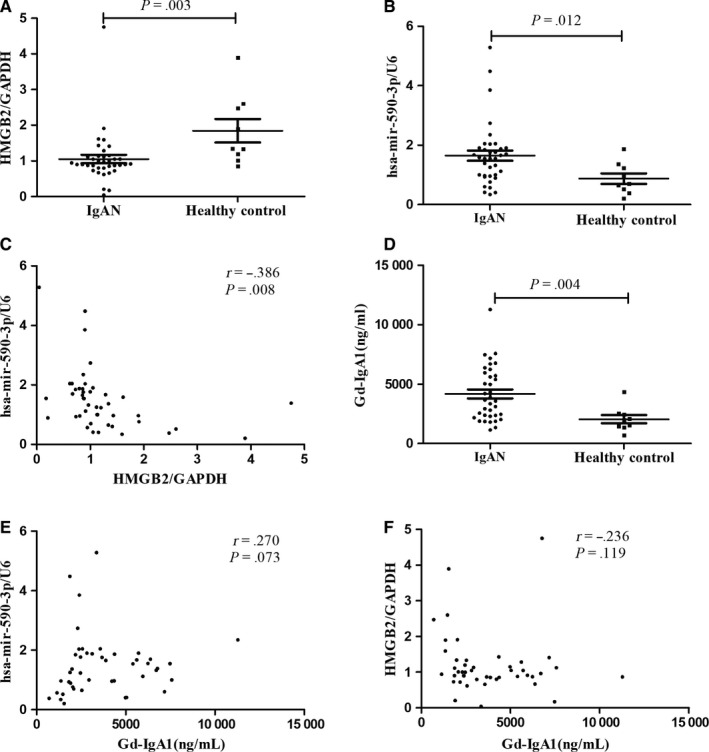
Compared with healthy controls, patients with IgAN presented significantly lower HMGB2 expression (A: 0.91 (0.80, 1.13) vs 1.34 (1.10, 2.54), *P* = .003), and higher hsa‐mir‐590‐3p expression (B: 1.55 (0.96,1.90) vs 0.70(0.45,1.30), *P* = .012); The scatter plot showed significantly negative correlation between the expression of hsa‐mir‐590‐3p and HMGB2 (C: *r* = −0.386, *P* = .008); Serum Gd‐IgA1 levels were significantly elevated in IgAN patients (D: 3612.67(2310.67, 5883.02) vs 1981.79(1406.55, 2487.19) ng/ml, *P* = .004), and the slight correlation tendency was found with hsa‐mir‐590‐3p and HMGB2 expression (D: *r* = 0.270, *P* = .073; F: *r* = −0.236, *P* = .119) in the whole population

To validate the contribution of HMGB2 and has‐miR‐590‐3p in IgAN, 37 patients with IgAN and 9 healthy controls were recruited to detect the expression of hsa‐miR‐590‐3p and HMGB2 in PBMCs. In the IgAN patients (the baseline data are shown in Table 5), the expression of HMGB2 in PBMCs was significantly down‐regulated compared with that in the healthy controls (0.91 (0.80, 1.13) vs 1.34 (1.10, 2.54), *P* = .003, Figure 4A). Meanwhile, the expression of has‐miR‐590‐3p in PBMCs was significantly up‐regulated in the IgAN patients (1.55 (0.96, 1.90) vs 0.70 (0.45, 1.30), *P* = .012, Figure 4B). Moreover, we also found a significant negative correlation between the expression of HMGB2 and that of hsa‐miR‐590‐3p in the whole population of IgAN patients and healthy controls (*r* = −0.386, *P* = .008, shown in Figure 4C).

**Table 5:** The baseline data of 37 patients with IgAN

Characters	Mean ± SD or median(IQR) or n
Male/female	23/14
Age (year)	42.11 ± 14.80
With hypertension (%)	26(70.27%)
SBP (mmHg)	135.97 ± 17.61
DBP (mmHg)	86.00 ± 10.39
24 h proteinuria (g/d)	1.75 (0.75,4.09)
SCR (μmol/L)	117 (82,189.50)
BUN (mmol/L)	6.60 (5.00,11.61)
Uric Acid (μmol/L)	342.19 ± 82.37
Albumin (g/L)	35.23 ± 8.98
TCHO (mmol/L)	4.40 (3.59,5.43)
TG (mmol/L)	1.93 (1.17, 2.60)
C3 (mg/L)	1.21 (1.04,1.38)
C4 (mg/L)	0.30 (0.25, 0.34)
Blood cell count in urine	24.50 (5.25,48.75)
Oxford classification (n)	
M score (M0/M1)	28/9
E score (E0/E1)	29/8
S score (S0/S1)	16/21
C score(C0/C1/C2)	17/18/2
T score (T0/T1/T2)	20/8/9

### 3.4 Increased plasma Gd‐IgA1levels and correlation with hsa‐miR‐590‐3p as well as HMGB2 expression in IgAN patients

Compared with healthy controls, patients with IgAN showed significantly higher levels of plasma Gd‐IgA1 (3612.67 (2310.67, 5883.02) vs 1981.79 (1406.55, 2487.19) ng/mL, *P* = .004; Figure 4D). Furthermore, the slight correlation tendency was found between serum Gd‐IgA1 levels and hsa‐miR‐590‐3p as well as HMGB2 expression (*r* = 0.270, *P* = .073, Figure 4E; *r* = −0.236, *P* = .119, Figure 4F) in the whole population.

### 3.5 HMGB2 was correlated with the severity of IgAN

**Figure 5:**
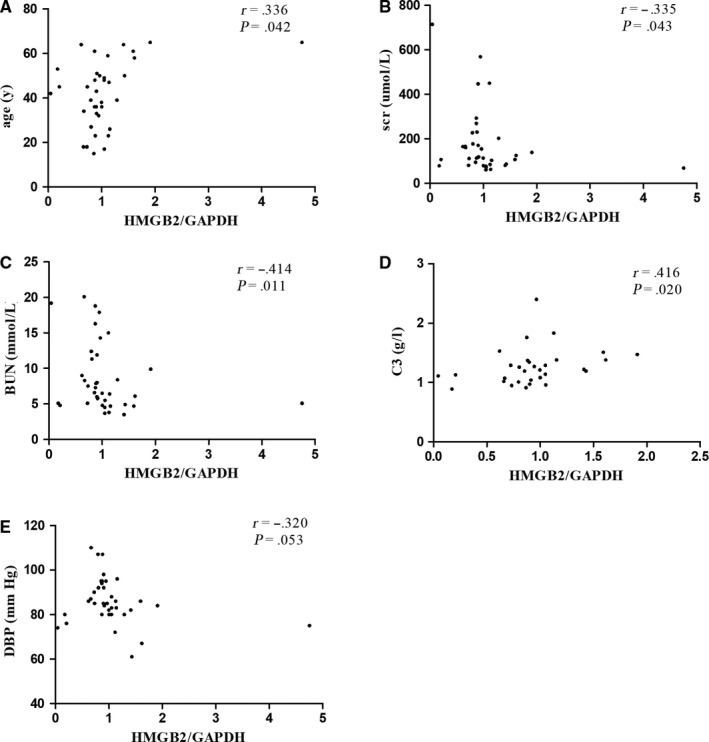
The expression levels of HMGB2 were significant positively correlated with age and serum C3(age: *r* = 0.336, *P* = .042; serum C3: *r* = 0.416, *P* = .020; as shown in A, D), but negatively correlated with serum creatinine, BUN, diastolic blood pressure(serum creatinine: *r* = −0.335, *P* = .043; BUN: *r* = −0.414, *P* = .011; as shown in B, C); and had the trend of negative correlation with diastolic blood pressure(*r* = −0.320, *P* = .053, E)

After the validation of the decreased expression of HMGB2 in IgAN patients, which was down‐regulated by the increased has‐miR‐590‐3p levels, we further explored their association with clinical findings and the pathological lesions in patients with IgAN. In the patients with IgAN, the expression of HMGB2 showed a significantly positive correlation with age and serum C3 levels (age: *r* = 0.336, *P* = .042; serum C3: *r* = 0.416, *P* = .020; Figure 5A, 5) and a significantly negative correlation with the serum creatinine, serum BUN (serum creatinine: *r* = −0.335, *P* = .043; BUN: *r* = −0.414, *P* = .011; Figure 5B, 5). Meanwhile, *HMGB2* expression also had a negative correlation trend with diastolic blood pressure (*r* = −0.320, *P* = .053; Figure 5E). These results indicate that lower *HMGB2* expression correlated with more severe clinical manifestations of IgAN. However, has‐miR‐590‐3p expression was not found to be correlated with the clinical or pathological manifestations of IgAN patients.

## 4 DISCUSSION

IgAN is a complex multifactorial disease with an unclear pathogenic mechanism. For the first time, we integrated the published microarray data from PBMCs to find more clues to uncover the pathogenesis of IgAN.

In the present study, a total of 19 DEGs (all down‐regulated) were identified through the comparison between the IgAN patients and the healthy controls in GEO datasets. The DEGs and differentially expressed miRNAs were mainly mapped to 5 IgAN‐associated terms (Figure 2). Among them, ‘defense to response to bacteria’ and ‘cell surface receptor signaling pathway’ were down‐regulated; however, ‘inflammatory response to antigenic stimulus’, ‘protein N‐linked glycosylation’ and ‘somatic diversification of immune receptors’ were up‐regulated. These results suggest that the immune system, inflammatory response and N‐glycosylation modifications were associated with IgAN. As seen in patients with IgA nephropathy, the external defense system to bacteria was weakened; on the other hand, once stimulated by an antigen, the responses of the immune system and the inflammatory system were enhanced, which was consistent with the previously reported results of the genome‐wide association studies (GWAS) of IgAN.^35^, ^36^ Moreover, although O‐linked glycosylation has been widely accepted to be a key step in the initiation of IgAN, a few studies have indicated that N‐linked glycosylation is also an important factor in the biological properties of IgA1.^37^, ^38^ There were a total of 6 genes and 2 miRNAs in the 5 terms. Both GCC2‐ and RAB1A‐encoded proteins are required for transport from endosomes to the Golgi.^39^ Moreover, RAB1A was reported to act as an oncogene to regulate cellular proliferation, growth, invasion and metastasis via the activation of the mTORC1 pathway in triple‐negative breast cancer.^40^ MAN1A1 encodes a class I mammalian Golgi 1,2‐mannosidase to catalyze the hydrolysis of three‐terminal mannose residues from peptide‐bound Man(9)‐GlcNAc(2) oligosaccharides.^41^ The NAMPT‐encoded protein is thought to be involved in many important biological processes, including metabolism, stress response and aging.^42^ The protein encoded by RASA1 is associated with cellular proliferation and differentiation.^43^ HMGB2, which has a high degree of similarity to HMGB1, was reported to be associated with cell viability, invasion and the chemotherapy resistance of glioblastoma and was reported to have antimicrobial activity.^44^, ^45^ Little is known about hsa‐miR‐648, and hsa‐miR‐590‐3p was reported to be related to Lynch syndrome in a published research paper.^46^

From the IgAN‐associated DEG‐miRNA network, it is clear that HMGB2 was in the core of the regulation network; the interaction between hsa‐590‐3p and HMGB2 was verified by the luciferase reporter assay and was validated in our IgAN cohort, in which miRNA‐hsa‐590‐3p and HMGB2 showed a significant negative correlation. In fact, we also explored the HMGB2 protein levels in 5 patients with IgAN and 7 healthy controls, and found the HMGB2 expression levels in PBMCs were significantly lower in patients than healthy controls, which was consistent with our previous results (Figure S1A, 1B). These findings suggest that HMGB2, which is targeted by hsa‐miR‐590‐3p, may be associated with the pathogenesis of IgA nephropathy.

The high‐mobility group box (HMGB) family consists of HMGB1‐HMGB4, which are related to inflammatory diseases by binding to DNA; these proteins induce large‐angle DNA bends, enhance the flexibility of DNA and perform numerous important biological processes. HMGB1 is secreted by the cells once it is stimulated by an antigen, and the secreted HMGB1 activates the innate immune system by binding to the cell surface receptors on various cell types.^47^ HMGB2 and HMGB1 are almost identical to each other and have often explored in the same study.^48^ Previous studies have shown that HMGB2 and HMGB1 share some functions, including the control of apoptosis by regulating the transcriptional activity of different members of the p53 family.^49^ Since HMGB1 is a multifunctional alarmin that drives autoimmune and inflammatory diseases, HMGB2 may also have a similar effect in IgAN. Serum complement 3 is one of the most important components of natural immunity, our result showed it had significantly positive correlation with HMGB2 expression levels, indicating HMGB2 may be involved the pathogenesis of IgAN by influencing the natural immunity system of patients. Robert Küchler et al and Takaishi H et al proved that HMGB2 has the function of antimicrobial activity against different commensal and pathogenic bacteria in the intestinal tract,^45^, ^50^ which has been proven to influence the pathogenesis of IgAN^51^; at the same time, this finding is consistent with our results in the present study. In addition, the expression level of HMGB2 was found to be related to the severity of IgAN, and both HMGB2 and hsa‐miR‐590‐3p expression levels had a correlation tendency with serum Gd‐IgA1, indicating that the elevated expression of hsa‐miR‐590‐3p down‐regulates HMGB2 expression and participates in the pathogenesis of IgAN. However, our study based on PBMCs, which include a range of different cells all with different immunological functions, different cells may had different expression levels of HMGB2 and hsa‐miR‐590‐3p, it is better to sort the PBMCs into subsets and then examine HMGB2 and hsa‐miR‐590‐3p expression levels and study the functions in each cell subset; what is more, the molecular mechanism by which HMGB2 regulates downstream factors to participate in IgAN is still unclear and requires further research.

In conclusion, we used bioinformatic analyses to analyse the published microarray data from PBMCs, and for the first time, we verified HMGB2 as the target gene of hsa‐miR‐590‐3p; we also identified that HMGB2 was correlated with the severity of IgAN, which provides more clues about the pathogenesis of IgAN.

## CONFLICT OF INTEREST

The authors declared that there is no conflict of interest regarding the publication of this paper.

## AUTHOR CONTRIBUTION

Conceived and designed the experiments: Yaling Zhai and Zhanzheng Zhao; performed experiments: Yanna Dou and Xiaoqing Long; analysed the data: Yuanyuan Qi and Genyang Cheng; contributed reagents/materials/analysis tools: Jing Xiao, Dong Liu and Zhangsuo Liu; wrote the paper: Yaling Zhai. All authors read and approved the final manuscript.

## Data Availability

Raw data used during the current study are available from the corresponding author on reasonable request for non‐commercial use.
