# Supplementary figures and images for: Elevated hsa‐miR‐590‐3p expression down‐regulates HMGB2 expression and contributes to the severity of IgA nephropathy
Source: J Cell Mol Med. 2019 Sep 26;23(11):7299–309. doi: 10.1111/jcmm.14582 (PMC6815813; doi:10.1111/jcmm.14582)

## Slide 1
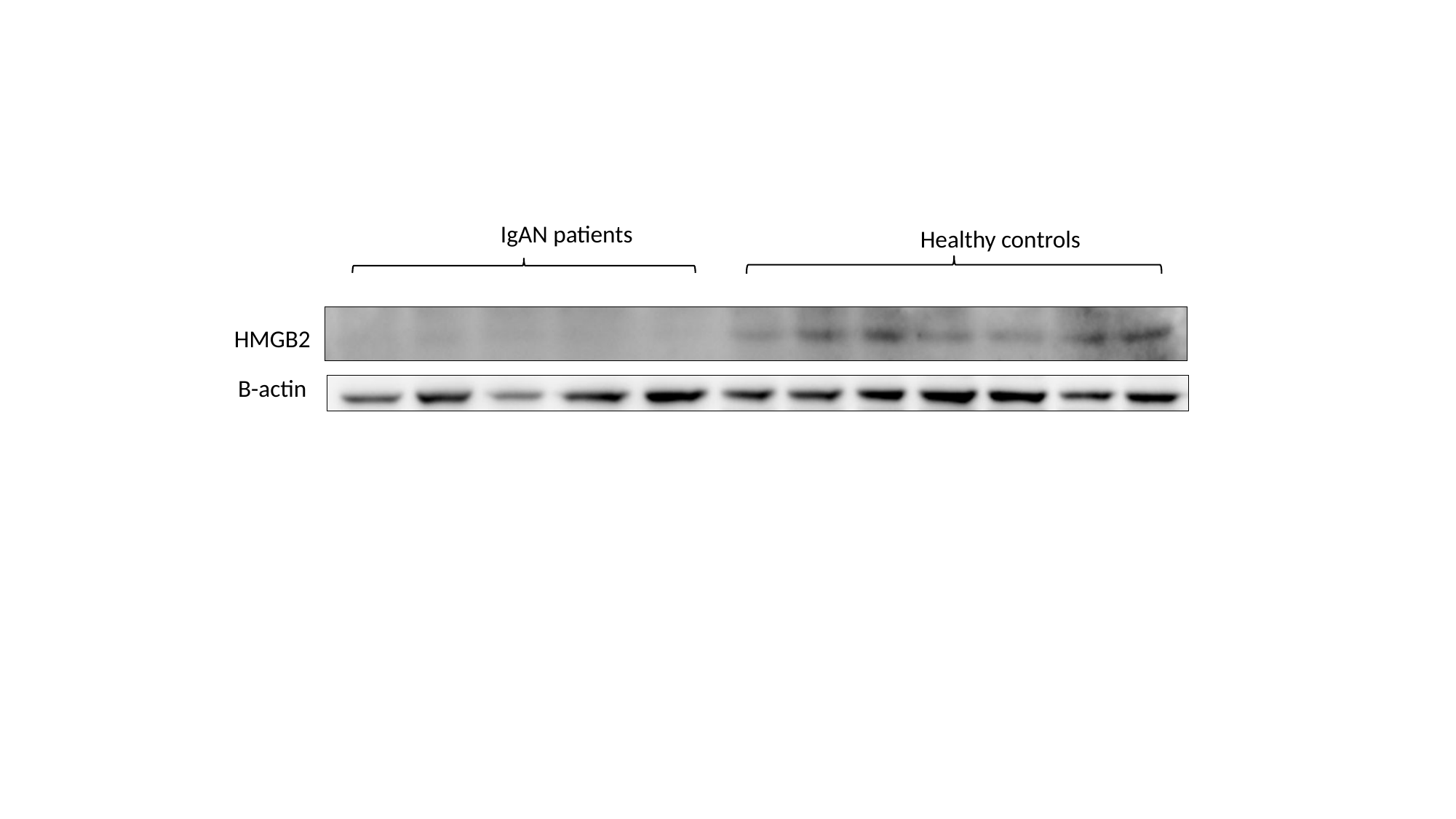

IgAN patients
Healthy controls
HMGB2
Β-actin

Supplement: Supplementary file 1 [file JCMM-23-7299-s001.pptx]

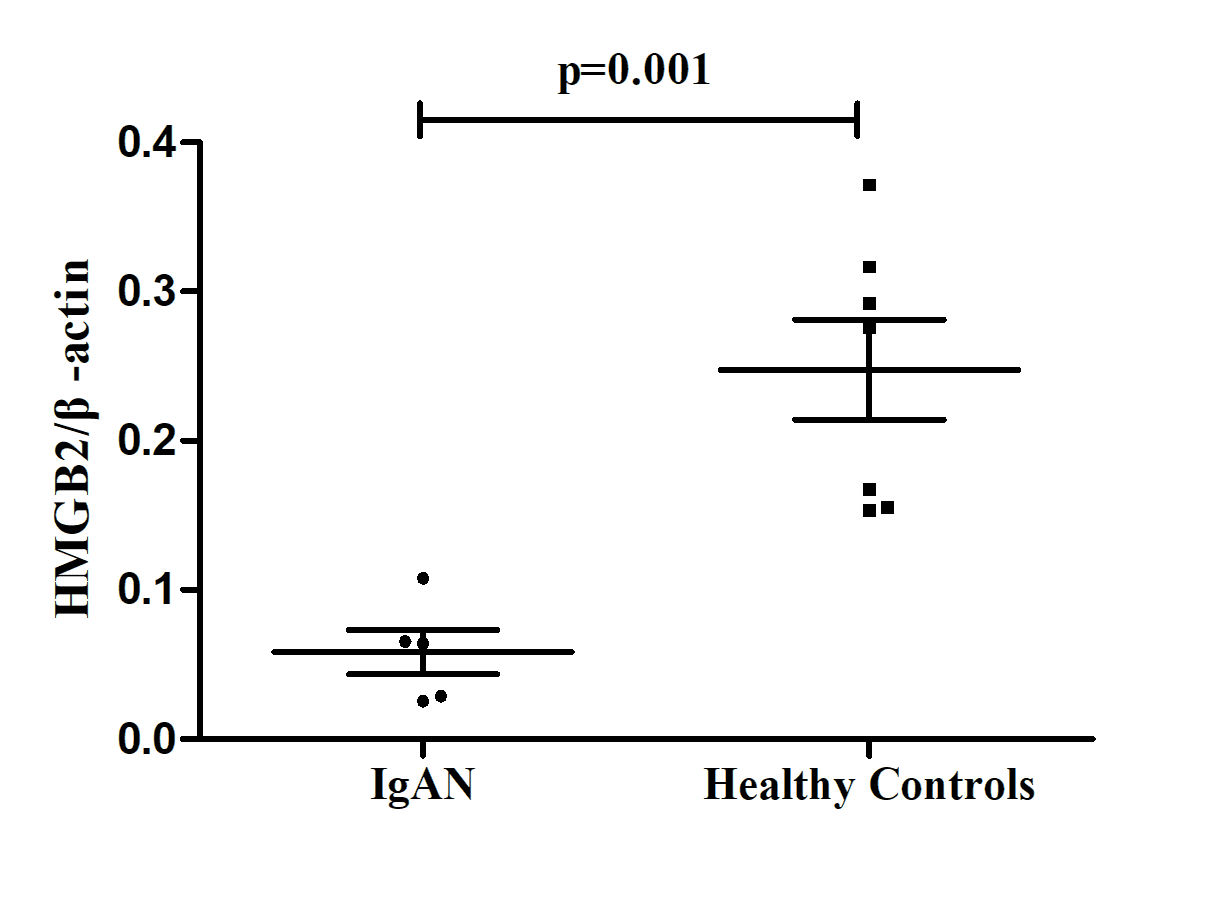

Supplement: Supplementary file 2 [file JCMM-23-7299-s002.tif]
